# Cause of death among patients with colorectal cancer: a population-based study in the United States

**DOI:** 10.18632/aging.104022

**Published:** 2020-11-28

**Authors:** Jiayuan Chen, Yongqiang Zheng, Haihong Wang, Dejun Zhang, Lei Zhao, Dandan Yu, Zhenyu Lin, Tao Zhang

**Affiliations:** 1 Cancer Center, Union Hospital, Tongji Medical College, Huazhong University of Science and Technology, Wuhan 430022, China; 2 State Key Laboratory of Oncology in South China, Sun Yat-Sen University Cancer Center, Sun Yat-Sen University, Guangzhou 510060, China

**Keywords:** colorectal cancer, cancer survivorship, cause of death, surveillance, epidemiology

## Abstract

CRC (Colorectal cancer) is one of the most common causes of death worldwide and in the US (United States). In this study, we aim to perform a population-based analysis on the cause of death among patients with CRC in the US. A total of 834,510 CRC patients diagnosed between 1975 and 2016 in the US were selected from the SEER (Surveillance, Epidemiology, and End Results) program. Causes of death among CRC patients were characterized and SMRs (standardized mortality ratios) of death from non-cancer causes were calculated. Among all CRC patients included in this study, a total of 531,507 deaths were recorded, of which 51.3% were due to CRC, 10.3% were due to other cancers, and 38.4% were due to non-cancer causes. Recently, there has been a relative decrease in index-cancer deaths and an increase in non-cancer causes among CRC patients. The mortality risk from non-cancer rises with accumulating age and longer follow-up time. Cardiovascular diseases are the most prevalent non-cancer causes, accounting for 20.3% of all deaths among CRC patients. Compared with the general population, the mortality rate of non-cancer deaths among CRC patients is doubled (SMR, 2.02; 95% confidence interval, 2.01-2.03).

## INTRODUCTION

CRC (Colorectal cancer) is the second most common cause of cancer-related death worldwide [1] and the third in the US [2]. Worldwide, in 2018, CRC killed 861,663 people [1]. In the US, during 2018, CRC was responsible for approximately 50,630 deaths [2].

In recent years, significant improvements have been made in the prevention, diagnosis, and treatment of CRC [3], resulting in a continuously improved survival. For patients with CRC in the United States, the relative 5-year survival is nearly 65% [4, 5]. As survivorship of CRC continues to increase [6–8], those involved in healthcare should aim to identify factors increasing the risk of death, which would help identify cancer patients at the highest risk of dying.

In this work, we aimed to characterize the causes of death among patients with CRC in the United States during a long-term follow-up time of more than 40 years. We also analyzed the causes of death among CRC patients as a function of calendar year, age at cancer diagnosis and time after diagnosis. Our work provides a contemporary resource for oncologists and PCPs (primary care physicians) as we highlight both major causes of death and basic clinical presentations (e.g., calendar year, age at cancer diagnosis and follow-up time after cancer diagnosis) among CRC patients which together may influence health care, patient-level decisions.

## RESULTS

Using data from the SEER (Surveillance, Epidemiology, and End Results) program, a total of 834,510 patients diagnosed with CRC between 1975 and 2016 were included in this study. Median follow-up time was 3.7 years (range: 0 to 41.9 years). The baseline characteristics of CRC patients are shown in Table 1. The mean age at diagnosis for all patients with CRC was 67.2 years. The majority of the patients were elderly (> 60 of age: 71.4%) and White (80.9%). During the entire follow-up, a total of 531,507 deaths were recorded. Among all the deaths, 51.3% were due to the index cancer (i.e. the CRC originally diagnosed in the patient), 10.3% were due to other cancers (i.e. a second primary cancer), and 38.4% were due to non-cancer causes (i.e. deaths from any medical cause other than cancer) (Table 2). Cardiovascular diseases caused the largest number of deaths among all non-cancer causes, accounting for over 20% of all deaths in patients with CRC.

**Table 1:** Characteristics of patients diagnosed with colorectal cancer between 1975 and 2016 in SEER 18 registries.

**Characteristics**	**No. of patients (%)**	**Person-years of follow-up**	**No. of deaths (%)**	**Non-cancer deaths**	
				**No. of observed deaths (%)**	**SMR^1^ (95% CI)**
All	834,510 (100%)	5,055,991	531,507 (100%)	203,990 (100%)	2.02 (2.01-2.03)
Age at diagnosis					
0-19	809 (0.1%)	4,665	170 (0.03%)	15 (0.01%)	6.18 (3.72-10.3)
20-39	22,855 (2.7%)	177,196	8,187 (1.5%)	744 (0.4%)	3.38 (3.15-3.64)
40-59	215,379 (25.8%)	1,634,525	91,560 (17.2%)	18,357 (9.0%)	2.70 (2.67-2.74)
60-79	426,178 (51.1%)	2,694,377	285,297 (53.7%)	115,513 (56.6%)	2.40 (2.38-2.41)
80+	169,289 (20.3%)	545,228	146,293 (27.5%)	69,361 (34.0%)	1.52 (1.51-1.53)
Sex					
Female	406,704 (48.7%)	2,493,868	259,344 (48.8%)	101,566 (49.8%)	2.10 (2.09-2.11)
Male	427,806 (51.3%)	2,562,123	272,163 (51.2%)	102,424 (50.2%)	1.95 (1.94-1.97)
Race					
White	675,249 (80.9%)	4,143,177	441,802 (83.1%)	174,670 (85.6%)	2.01 (2.00-2.02)
Black	89,262 (10.7%)	472,527	55,525 (10.4%)	17,839 (8.7%)	1.94 (1.91-1.97)
Other	69,999 (8.4%)	440,287	34,180 (6.4%)	11,481 (5.6%)	2.49 (2.44-2.53)
Year of diagnosis					
1975-1989	151,952 (18.2%)	1,267,407	144,324 (27.2%)	63,546 (31.2%)	2.34 (2.32-2.36)
1990-1999	141,277 (16.9%)	1,171,883	116,398 (21.9%)	52,005 (25.5%)	2.15 (2.14-2.17)
2000-2009	326,362 (39.1%)	2,098,941	201,504 (37.9%)	72,762 (35.7%)	1.81 (1.79-1.82)
2010-2016	214,919 (25.8%)	517,760	69,281 (13.0%)	15,677 (7.7%)	1.70 (1.67-1.72)
Marital status					
Married	452,732 (54.3%)	3,148,549	271,835 (51.1%)	101,673 (49.8%)	1.95 (1.94-1.96)
Unmarried	339,099 (40.6%)	1,661,754	238,270 (44.8%)	93,497 (45.8%)	2.11 (2.10-2.12)
Unknown	42,679 (5.1%)	245,688	21,402 (4.0%)	8,820 (4.3%)	2.02 (1.97-2.06)
Stage					
In situ	38,726 (4.6%)	390,706	19,398 (3.6%)	14,488 (7.1%)	2.19 (2.15-2.23)
Localized	297,373 (35.6%)	2,456,175	156,479 (29.4%)	98,197 (48.1%)	1.97 (1.96-1.98)
Regional	271,069 (32.5%)	1,783,020	177,373 (33.4%)	68,573 (33.6%)	1.91 (1.90-1.93)
Distant	155,377 (18.6%)	266,283	141,487 (26.6%)	11,112 (5.4%)	2.46 (2.41-2.51)
Unstaged	71,965 (8.6%)	159,807	36,770 (6.9%)	11,620 (5.7%)	2.89 (2.84-2.94)
Surgery					
Yes	715,101 (85.7%)	4,840,935	431,581 (81.2%)	186,280 (91.3%)	1.95 (1.94-1.96)
No	110,264 (13.2%)	189,800	92,843 (17.5%)	16,129 (7.9%)	3.47 (3.42-3.52)
Unknown	9,145 (1.1%)	25,257	7,083 (1.3%)	1,581 (0.8%)	2.76 (2.63-2.90)
Chemotherapy					
Yes	238,583 (28.6%)	1,215,176	139,652 (26.3%)	23,589 (11.6%)	1.62 (1.60-1.64)
No/Unknown	595,927 (71.4%)	3,840,815	391,855 (73.7%)	180,401 (88.4%)	2.09 (2.08-2.10)
Radiotherapy					
Yes	97,217 (11.6%)	548,483	57,892 (10.9%)	13,042 (6.4%)	1.84 (1.81-1.88)
No/unknown	737,293 (88.4%)	4,507,508	473,615 (89.1%)	190,948 (93.6%)	2.04 (2.03-2.05)
Site					
Cecum	126,941 (15.2%)	698,483	87,405 (16.4%)	34,823 (17.1%)	1.88 (1.86-1.90)
Appendix	12,526 (1.5%)	66,680	4,769 (0.9%)	1,028 (0.5%)	1.83 (1.72-1.94)
Ascending colon	95,882 (11.5%)	531,641	60,438 (11.4%)	26,269 (12.9%)	1.90 (1.88-1.92)
Hepatic flexure	27,550 (3.3%)	156,177	18,758 (3.5%)	7,638 (3.7%)	1.96 (1.92-2.01)
Transverse colon	50,104 (6.0%)	284,944	33,377 (6.3%)	13,862 (6.8%)	2.08 (2.05-2.12)
Splenic flexure	19,854 (2.4%)	117,130	13,483 (2.5%)	4,934 (2.4%)	2.03 (1.98-2.09)
Descending colon	37,296 (4.5%)	244,856	24,022 (4.5%)	9,980 (4.9%)	2.19 (2.15-2.23)
Sigmoid colon	185,664 (22.2%)	1,295,333	115,320 (21.7%)	48,365 (23.7%)	2.09 (2.07-2.11)
Large intestine, NOS	29,555 (3.5%)	469,411	24,401 (4.6%)	16,727 (8.2%)	2.09 (2.06-2.12)
Rectosigmoid junction	71,794 (8.6%)	90,971	46,862 (8.8%)	4,983 (2.4%)	2.45 (2.38-2.52)
Rectum	177,344 (21.3%)	1,100,366	102,672 (19.3%)	35,381 (17.3%)	2.06 (2.03-2.08)

Abbreviations: SMR, standardized mortality ratios; CI, confidence interval.

^1^ The SMRs were estimated as the ratios of observed to expected number of deaths. The observed values represented the number of deaths in cancer patients, whereas the expected values represented the number of individuals who died of the same causes in the general population, with a similar distribution of age, sex, race, and calendar year.

The mortality rate of all non-cancer deaths was 2.02 (95% CI [confidence interval], 2.01-2.03) times that of the general cancer-free population with similar demographic distribution. The most frequent distant metastatic site in CRC was liver metastasis (14.8%), followed by lung metastasis (5%). Though less frequent, SMRs (standardized mortality ratios) were elevated for patients who were with distant metastases (Supplementary Tables 1–3). Compared to the cause-specific mortality in the general population, SMRs were the highest for renal diseases and infectious disease among all major causes, with an SMR of 2.47 (95% CI, 2.41-2.54) and 2.32 (95% CI, 2.28-2.35), respectively. Among less frequent causes, elevations in the SMR were remarkable for certain conditions originating in the perinatal period (SMR, 124.5; 95% CI, 59.3-261.1), and complications of pregnancy, childbirth and puerperium (SMR, 20.6; 95% CI, 14.6-29.0). This is because the mortality rates from these causes are particularly low in the general population. Detailed causes of death for CRC patients are presented in Table 2. For most causes, the SMR were higher for patients with advanced diseases, not receiving surgery, chemotherapy or radiotherapy (Table 1 and Supplementary Tables 4–7).

**Table 2:** Causes of death for patients diagnosed with colorectal cancers between 1975 and 2016 in SEER 18 registries.

**Causes of death**	**Patients with colorectal cancer**			**General population^1^**		**SMR^1,2^ (95% CI)**
	**No. of observed deaths (%)**	**Mortality rates (per 100,000 person-years)**		**No. of expected deaths (%)**	**Mortality rates (per 100,000 person-years)**	
All causes of death	531,507 (100.0%)	10,512.4		NA	NA	NA
Colorectal cancer	272,901 (51.3%)	5,397.6		NA	NA	NA
Other cancers	54,616 (10.3%)	1,080.2		NA	NA	NA
Non-cancer causes	203,990 (38.4%)	4,034.6		100,833.4	1,994.3	2.02 (2.01 -2.03)
Infectious diseases	14,942 (2.8%)	295.5		6,453.0	127.6	2.32 (2.28 -2.35)
Pneumonia and influenza	8,792 (1.7%)	173.9		3,978.9	78.7	2.21 (2.16 -2.26)
Syphilis	2 (0.0004%)	0.04		2.6	0.05	0.76 (0.19 -3.05)
Tuberculosis	75 (0.01%)	1.5		79.5	1.6	0.94 (0.75 -1.18)
Septicemia	4,207 (0.8%)	83.2		1,505.5	29.8	2.79 (2.71 -2.88)
Other infectious and parasitic diseases including HIV	1,866 (0.4%)	36.9		659.8	13.0	2.83 (2.70 -2.96)
Cardiovascular diseases	108,086 (20.3%)	2,137.8		57,753.6	1,142.3	1.87 (1.86 -1.88)
Diseases of heart	82,947 (15.6%)	1,640.6		44,470.3	879.6	1.87 (1.85 -1.88)
Hypertension without heart disease	2,820 (0.5%)	55.8		995.9	19.7	2.83 (2.73 -2.94)
Aortic aneurysm and dissection	1,478 (0.3%)	29.2		997.1	19.7	1.48 (1.41 -1.56)
Atherosclerosis	1,961 (0.4%)	38.8		1,023.8	20.2	1.92 (1.83 -2.00)
Cerebrovascular diseases	17,750 (3.3%)	351.1		9,690.2	191.7	1.83 (1.81 -1.86)
Other diseases of arteries, arterioles, capillaries	1,130 (0.2%)	22.3		574.8	11.4	1.97 (1.85 -2.08)
Respiratory diseases	13,713 (2.6%)	271.2		7,031.0	139.1	1.95 (1.92 -1.98)
Chronic obstructive pulmonary disease and allied cond	13,713 (2.6%)	271.2		7,031.0	139.1	1.95 (1.92 -1.98)
Gastrointestinal diseases	2,907 (0.5%)	57.5		1,719.9	34.0	1.69 (1.63 -1.75)
Stomach and duodenal ulcers	618 (0.1%)	12.2		314.9	6.2	1.96 (1.81 -2.12)
Chronic liver disease and cirrhosis	2,289 (0.4%)	45.3		1,404.5	27.8	1.63 (1.56 -1.70)
Renal diseases	4,919 (0.9%)	97.3		1,988.9	39.3	2.47 (2.41 -2.54)
Nephritis, nephrotic syndrome and nephrosis	4,919 (0.9%)	97.3		1,988.9	39.3	2.47 (2.41 -2.54)
External injuries	6,646 (1.3%)	131.4		4,257.7	84.2	1.56 (1.52 -1.60)
Accidents and adverse effects	5,118 (1.0%)	101.2		3,169.6	62.7	1.61 (1.57 -1.66)
Suicide and self-inflicted injury	1,360 (0.3%)	26.9		859.1	17.0	1.58 (1.50 -1.67)
Homicide and legal intervention	168 (0.0%)	3.3		227.9	4.5	0.74 (0.63 -0.86)
Other non-cancer causes	52,777 (9.9%)	1,043.9		21,629.1	427.8	2.44 (2.42 -2.46)
Alzheimer’s disease	7,533 (1.4%)	149.0		2,432.7	48.1	3.10 (3.03 -3.17)
Diabetes mellitus	7,249 (1.4%)	143.4		3,676.3	72.7	1.97 (1.93 -2.02)
Congenital anomalies	174 (0.03%)	3.4		129.7	2.6	1.34 (1.16 -1.56)
Certain conditions originating in perinatal period	7 (0.001%)	0.1		0.1	0.001	124.5 (59.3 -261.1)
Complications of pregnancy, childbirth, puerperium	33 (0.01%)	0.7		1.6	0.03	20.58 (14.63 -28.95)
Symptoms, signs and ill-defined conditions	2,582 (0.5%)	51.1		1,291.2	25.5	2.00 (1.92 -2.08)
Other cause of death	35,199 (6.6%)	696.2		14,093.9	278.8	2.50 (2.47 -2.52)

Abbreviations: SMR, standardized mortality ratios; CI, confidence interval.

^1^The calculating of SMR is based on the hypothesis that the general population is cancer-free, thus the expected deaths and SMR for colorectal cancer and other cancers cannot be calculated.

^2^The SMRs were estimated as the ratios of observed to expected number of deaths. The observed values represented the number of deaths in cancer patients, whereas the expected values represented the number of individuals who died of the same causes in the general population, with a similar distribution of age, sex, race, and calendar year.

### Objective 1: Cause of death in patients with CRC per calendar year

**Figure 1:**
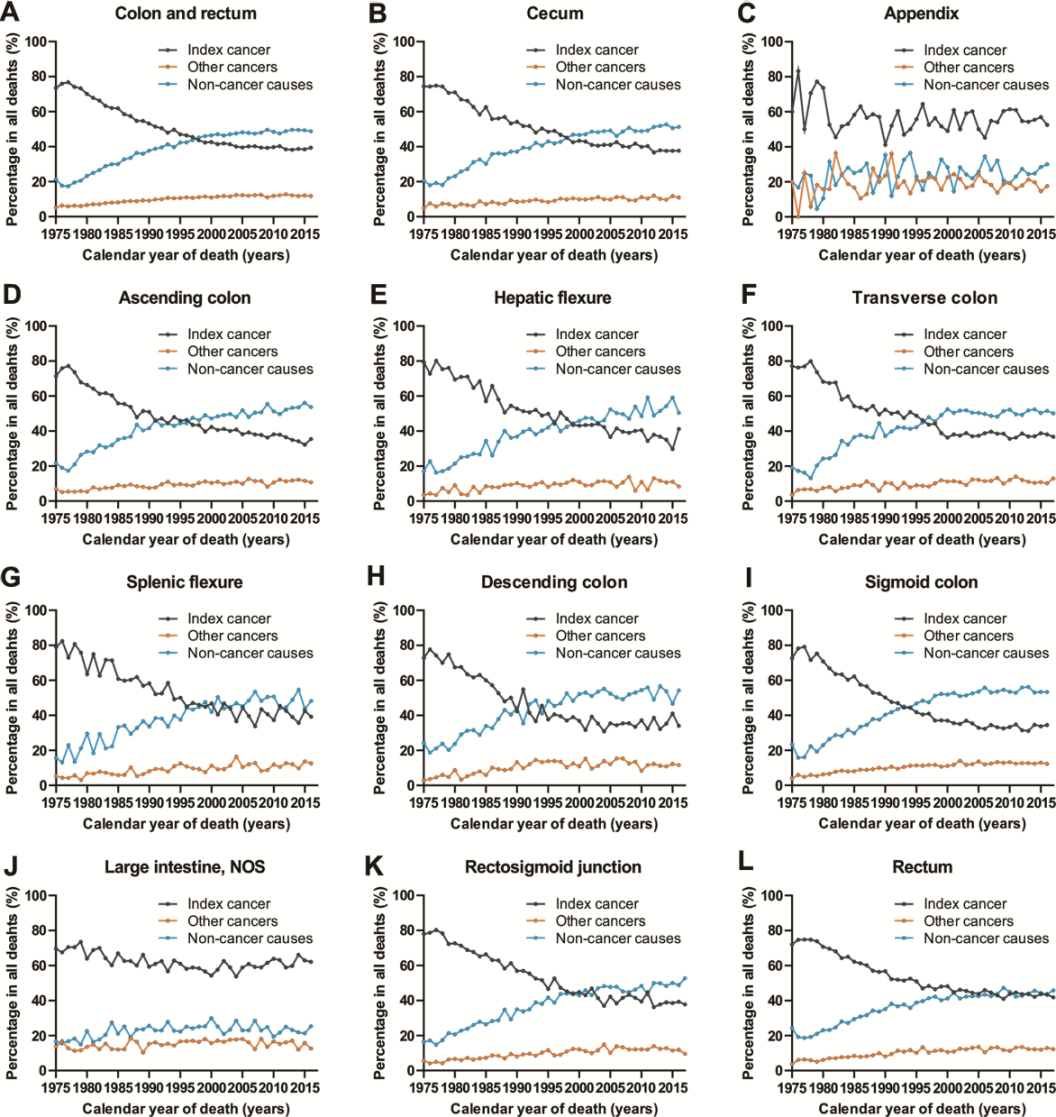
**Cause of death among patients diagnosed with CRC in SEER 9 registries by calendar year of death.** (**A**) Cause of death among patients with CRC by year of death; (**B**) cause of death among patients with cancer of cecum by year of death; (**C**) cause of death among patients with appendicular cancer by year of death; (**D**) cause of death among patients with cancer of ascending colon by year of death; (**E**) cause of death among patients with cancer of hepatic flexure by year of death; (**F**) cause of death among patients with cancer of transverse colon by year of death; (**G**) cause of death among patients with cancer of splenic flexure by year of death; (**H**) cause of death among patients with cancer of descending colon by year of death; (**I**) cause of death among patients with cancer of sigmoid colon by year of death; (**J**) cause of death among patients with cancer of large intestine, NOS by year of death; (**K**) cause of death among patients with cancer of rectosigmoid junction by year of death; (**L**) cause of death among patients with cancer of rectum by year of death.

We analyzed death trends in CRC patients by assessing deaths due to CRC (index cancer), deaths due to other cancers and deaths due to non-cancer causes among all CRC subtypes. Patients who are more likely to die from index cancer are those with cancers of the appendix and large intestine, NOS, cancer types for which the cancer-specific prognosis has been relatively stable for the past few decades (Figure 1). With the improvement in cancer prognosis (cecum, ascending colon, hepatic flexure, transvers colon, splenic flexure, descending colon, segment colon, rectum and rectosigmoid junction), there appears to be a continuous increase in the number of deaths due to non-cancer causes, and a decrease in the number of deaths due to index cancer in cancer survivors (Figure 1). In 2016, non-cancer causes accounted for 48.8% of all deaths among all CRC patients, while the index cancer accounted for 39.4% of all deaths.

### Objective 2: Cause of death in patients with CRC by age at cancer diagnosis

**Figure 2:**
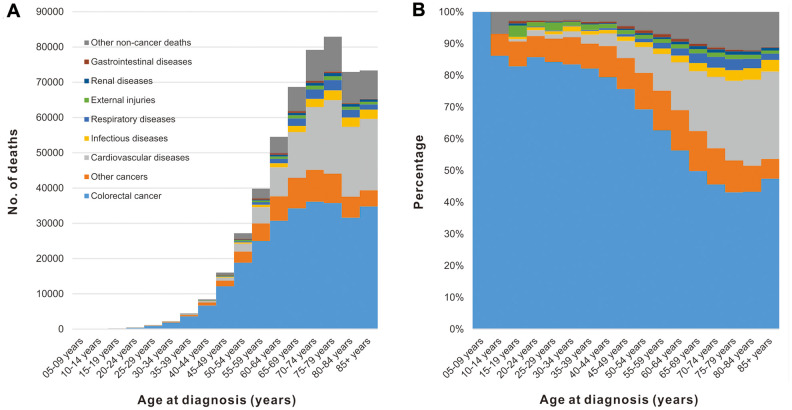
**Cause of death among patients diagnosed with CRC in SEER 18 registries by age at diagnosis.** (**A**) Number of deaths from different causes by age at diagnosis. (**B**) Percentage of deaths from different causes by age at diagnosis.

Among CRC patients, the number of deaths increased with age, reaching a peak at 70-75 years of age (Figure 2A). Although CRC was the major cause of death among all age groups, the percentage of patients dying from CRC decreased with increasing of age, while the non-cancer causes (specifically cardiovascular diseases) were increasing (Figure 2B).

**Figure 3:**
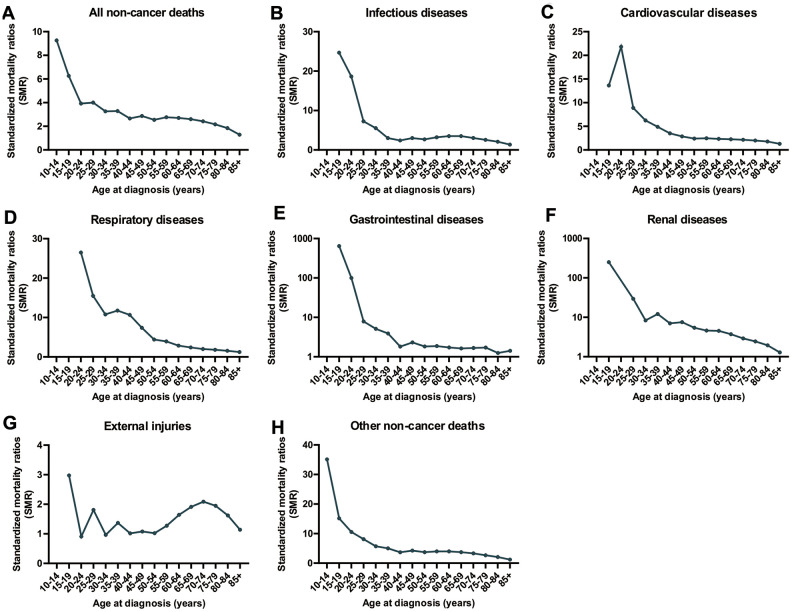
**SMRs of non-cancer deaths among patients diagnosed with CRC in SEER 18 registries by age at diagnosis.** (**A**) SMRs of all non-cancer deaths by age; (**B**) SMRs of infectious diseases by age; (**C**) SMRs of cardiovascular diseases by age; (**D**) SMRs of respiratory diseases by age; (**E**) SMRs of renal diseases by age; (**F**) SMRs of external injuries by age; (**G**) SMRs of cardiovascular diseases by age; (**H**) SMRs of other non-cancer deaths by age.

Compared to individuals of a similar age in the general US population, cancer patients of all ages have an increased risk of non-cancer deaths (Figure 3). For nearly all types of non-cancer causes, the highest SMR of death was observed in cancer patients younger than 40 years old. However, the prevalence of non-cancer deaths in CRC patients was very low with only 759 deaths in CRC patients younger than 40 years of age between 1975 and 2016 (Table 1). The SMR of non-cancer deaths among CRC patients gradually decreases with increasing age at cancer diagnosis. This decreasing trend was not observed in deaths caused by external injuries. In this context, another peak of SMR in CRC patients occurred at ages between 70 and 74 years old (Figure 3).

### Objective 3: Cause of death in patients with CRC by time after diagnosis

**Figure 4:**
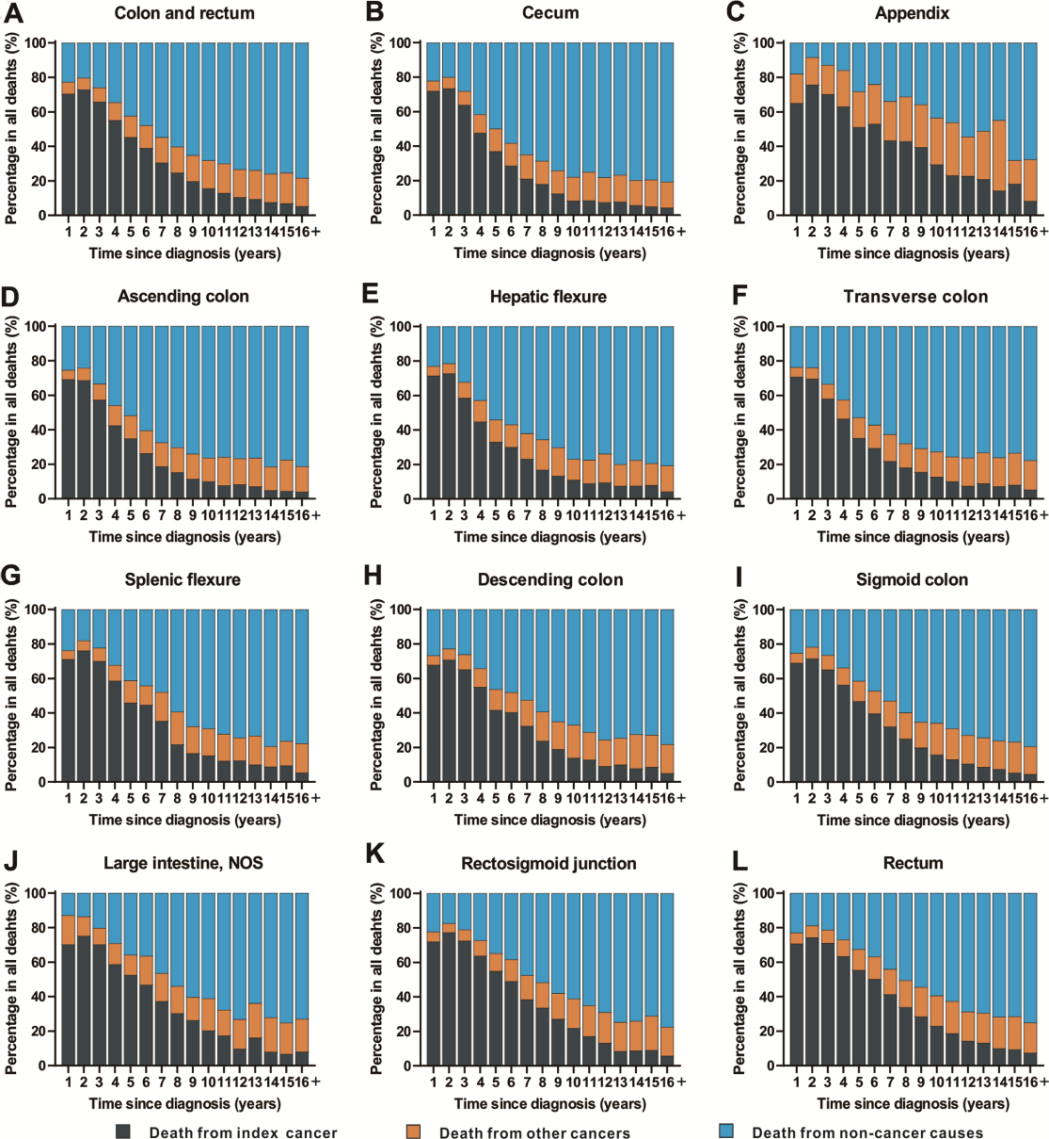
**Cause of death among patients diagnosed with CRC in SEER 18 registries by follow-up time from diagnosis.** (**A**) Cause of death among patients with CRC by time from diagnosis; (**B**) cause of death among patients with cancer of cecum by time from diagnosis; (**C**) cause of death among patients with appendicular cancer by time from diagnosis; (**D**) cause of death among patients with cancer of ascending colon by time from diagnosis; (**E**) cause of death among patients with cancer of hepatic flexure by time from diagnosis; (**F**) cause of death among patients with cancer of transverse colon by time from diagnosis; (**G**) cause of death among patients with cancer of splenic flexure by time from diagnosis; (**H**) cause of death among patients with cancer of descending colon by time from diagnosis; (**I**) cause of death among patients with cancer of sigmoid colon by time from diagnosis; (**J**) cause of death among patients with cancer of large intestine, NOS by time from diagnosis; (**K**) cause of death among patients with cancer of rectosigmoid junction by time from diagnosis; (**L**) cause of death among patients with cancer of rectum by time from diagnosis.

The time period following a cancer diagnosis (all sites) represents the period with the highest risk of cancer-specific mortality (Figure 4 and Supplementary Table 8). With an increasing period of time from the point of cancer diagnosis, there was an increasing trend in deaths from non-cancer causes. In fact, non-cancer causes became the leading cause of death in cancer survivors with a cancer history of more than 5 years (Figure 4 and Supplementary Table 8).

Compared with the general population, CRC patients displayed SMRs which were higher than the general population throughout the entire follow-up period (Supplementary Table 8). In the first 5-years after diagnosis, SMRs decreased slightly. An increasing trend for the SMR of non-cancer death was observed in cancer survivors with a cancer diagnosis of more than five years, and the highest SMR of non-cancer mortality was observed after a long-term follow-up of more than 15 years (Supplementary Table 8).

## DISCUSSION

In this study we present an analysis of cause of death among more than 0.8 million CRC patients. In patients diagnosed with CRC, cause of death varies as a function of calendar year of diagnosis, follow-up time, and other clinical variables, such as age and race. Non-cancer causes are the major health threats for long-term CRC survivors, and the mortality rate of non-cancer death is more than double in cancer patients compared to the general population. In recent years, with the increase in the number of long-term CRC survivors, non-cancer causes (especially cardiovascular diseases) have become the major cause of death in CRC patients, and are still showing an increasing trend. Previous studies have analyzed the causes of death among patients with different types of cancer [9, 10]. Further, several investigations have also reported the causes of death in specific cancer types such as breast cancer, and head and neck cancer [11, 12], or among a subgroup of patients such as adolescents and young adults [13]. Unfortunately, no detailed research has been performed on colorectal cancer. Thus, the work presented here is unique.

We found that deaths from non-cancer causes and second primary cancer were increasing in recent years, and non-cancer diseases are now the main cause of death among CRC patients. An increase in the risk of deaths from non-cancer causes and second cancer was observed when the follow-up time was accumulating. It is known that the advances in cancer treatments, although remarkably reduced mortality from the primary cancer, may increase the future risk of death from the causes other than the primary cancer, including development of a second primary cancer or other non-cancer comorbidities [12, 14, 15]. With better treatment patterns in palliative [16] and adjuvant [17–20] settings, the last 2 decades have seen remarkable improvements in the prognosis among patients with CRC in the US [21]. Systematic therapy for metastatic CRC is tailored with disease-specific and patient-specific predictive markers. With the progress in the research of tumorigenic mechanism, many potential therapy targets have been found [22–24], which contributed greatly to the prognosis of CRC patients. Paralleled with the breakthroughs in surgical and allied areas, the increasing kinds and number of effective drugs for CRC has led to considerable improvements of overall survival. Higher education status and healthy dietary can also improve the prognosis of CRC patients [25–27]. Thus, after a CRC diagnosis, patients may live longer to a stage when non-cancer causes could affect their survival time significantly. The Sweden cohort revealed that after the median follow-up, almost half of the CRC patients who were dead had died from other causes than colon cancer. Our study further demonstrated that non-cancer causes account for the majority of the deaths for the patients with a long CRC history [28]. It’s also reported that long-term CRC survivors are at high risk of developing a second primary malignancy [29]. With an increasing risk of second primary cancer by time from diagnosis, nearly 10% of CRC survivors will have a second primary cancer.

Our work also evaluated the cause of death among cancer patients as a function of age at diagnosis. We found that the proportion of non-cancer deaths, especially the cardiovascular diseases, were increasing with an increased age. Most patients with colorectal cancer are older and may have multiple comorbidities. In a study from Canada, the researchers found that cardiovascular diseases is the major non-cancer cause of death in the elderly CRC patients [30], consistent with our results. Age is one of the major risk factors associated with some non-cancer diseases, such as cardiovascular diseases and chronic obstructive pulmonary disease [31, 32]. Intriguingly, we found that patients younger than 40 posed a very high risk of death from non-cancer causes when comparing to the age-matched general population, which is agree with previous studies [10, 13]. This elevation may be due to the low mortality rate of deaths from non-cancer diseases in young individuals of the general cancer-free population, as these diseases are often related with elderly age.

Throughout all time periods, the most common non-cancer cause of death was cardiovascular diseases, mainly heart disease and cerebrovascular disease. The high risk of heart diseases in CRC patients may due to the cardiac side effects of systemic chemotherapy. Acute cardiotoxicity is recognized as a potentially severe adverse event of 5-FU and capecitabine treatment [33, 34]. This dose-dependent toxicity could lead to acute myocardial infarction and ischemia [35, 36]. A retrospective study of adjuvant chemotherapy revealed that there was no relevance between relative dose intensity and overall survival for stage III colon cancer [37], while another survey found better 5-year overall survival for patients who received >70% relative dose intensities [38]. Thus, the scientific evidence is too rare to guide clinical decisions on whether to treat patients with these drugs, but the risk of severe cardiotoxicity should be evaluated against the expected treatment benefits. In addition, a study of the Dutch Colorectal Cancer Group analyzed cardiotoxicity in 1973 patients who received capecitabine for metastatic colorectal cancer [39]. They reported that a significant number of patients suffered cardiotoxicity following treatment with capecitabine, and a high incidence of cardiac cases was observed in patients who were treated with the combination of capecitabine, oxaliplatin and bevacizumab. A few cases of oxaliplatin-induced arrhythmias have also been reported [40]. Further, the risk of cerebrovascular events was significantly increased among patients with advanced colorectal cancer who received bevacizumab [41], as bevacizumab may cause rare toxic effects such as cerebral hemorrhage, intracranial hemorrhage and subarachnoid hemorrhage [42]. Bevacizumab was also associated with higher risk of hypertension and arterial thrombotic events [43], which in turn increased the risk of cardiac ischemia (myocardial infarction or angina), stroke or transient ischemic attack.

Patients with CRC has a particularly increased mortality rate from infectious diseases. As a predominant cause of death among patients with cancer, fatal infections have been interpreted to be a consequence of the immunosuppression induced by the malignancy itself and by various modern cancer therapies, including chemotherapeutic regimens, invasive procedures, or medical devices and malnutrition [44–46]. Neutropenia-related infection has been shown to be a common side effect of chemotherapy and can result in sepsis [47, 48]. Further, patients undergoing monoclonal antibody therapy have also been shown to be at risk of developing immunosuppression and thus being at equal risk of infections. In cases of rapid tumor growth, insufficient blood supply can lead to a nidus of infection. The risk of infectious disease should be evaluated individually as it is dependent on the patients’ immune status, treatment methods and history of infections. Therefore, health practitioners should use preventive measures to reduce the risk of infections and select appropriate treatments.

Furthermore, our study showed that the SMR of suicide and self-inflicted injury were higher in the first year after diagnosis (Supplementary Table 3). The incidence of suicidal death increased substantially after the diagnosis of a cancer [49]. Depression, anxiety and opioid use have been linked with suicide among cancer patients. In patients with CRC, such issues are often linked with demands related to the care of ostomies [50]. In addition, stress may weaken the immune system, which could lead to poorer response to treatments and mental stress, which in turn add to the risk of a suicide attempt [51]. AD (Alzheimer’s disease) was also another important non-cancer cause of death among CRC patients (Supplementary Table 3). In recent years, some researchers have found that diet and constipation might influence the progression of AD [52]. Constipation is a growing health problem among CRC patients and such patients might have a higher risk of mortality from AD than the general US population.

This study has some limitations. First, there is a risk of reporting bias in death certificates which could lead to misclassification of causes of death [53, 54]. However, the SEER mortality data were provided by the NCHS (National Center for Health Statistics) and NVSS (National Vital Statistics System) and systematic and standardized data collection procedures are used to ensure that the causes of death recorded in SEER are accurate [55]. Further, previous studies have also examined the validity and reliability of the use of death certificates recorded in the SEER and the results suggested that they were acceptable [56, 57]. Second, SEER does not contain information regarding pre-existing comorbidities, performance status, quality of life, as well as detailed and complete information of cancer treatment. Thus, we were not able to analyze the cause and effect relationship between different risk factors and distinct causes of death. Nevertheless, analyzing the extensive amount of data available from the SEER database remains a powerful, useful, and integral tool in medical research for the purpose of exploratory analyses [55]. Third, we were unable to obtain data on genetic information (such as MMR (defective mismatch repair) genes or mutations status of KRAS and BRAF) and dietary habits, which prohibited us from taking these features into account.

In conclusion, the risk of index cancer death in CRC patients decreases with time, while that of non-cancer causes increases. Patients with CRC are at high risk of non-cancer death. In fact, the mortality rate of non-cancer death among CRC patients is twice that of the general population. The SMR of non-cancer deaths increases with longer follow-up time. CRC is the major cause of death in the first few years after a cancer diagnosis. Cardiovascular diseases are also important causes of death in CRC patients. Identifying the major causes of death among cancer patients is important for the formulation of health strategies. Investigating the influence of non-cancer deaths on the health outcomes of CRC patients is an important field for future research.

## MATERIALS AND METHODS

### Data source and study population

Using data from the SEER program, we performed a retrospective population-based cohort study. As a system of population-based cancer registries from the National Cancer Institute, the program routinely collects and reports data on cancer demographics, incidence, follow-up data, anatomic site, morphology, stage, therapy and socioeconomic status of cancer patents in the United States [58].

**Figure 5:**
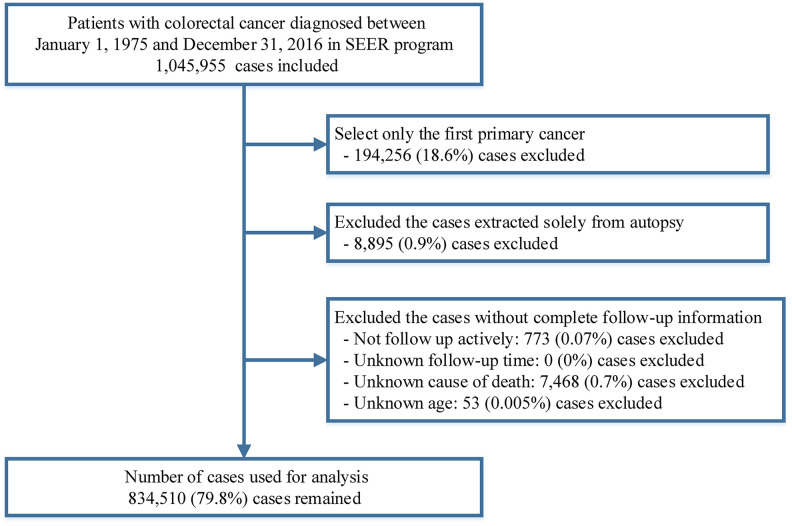
**Flow chart of case inclusion and exclusion criteria in this study.**

All patients diagnosed with CRC between 1975 and 2016 were extracted from the SEER 18 database (2019 submission) using SEER*Stat software version 8.3.6 [58]. All types of cancers excluding a histology of mesothelial neoplasms (ICD-O-3 [International Classification of Diseases for Oncology third edition] codes: 9050-9055), Kaposi sarcoma (ICD-O-3 codes: 9140) and hematological malignancies (ICD-O-3 codes: 9590-9992) diagnosed in the colon and rectum (ICD-O-3 site codes: C18.0-C18.9, C26.0) were included in this study. Only the first primary diagnosis of colorectal cancer was included. Patients were excluded if their diagnoses were obtained only from death certificates or autopsies. We further excluded patients without active follow-up and those with unknown follow-up time, age at diagnosis or cause of death. (Figure 5). To analyze the causes of death among CRC patients as a function of calendar year of death, we extracted death data of CRC patients from 1975 to 2016 using the SEER-9 incidence-based mortality session, which accurately records the year of death but not the year of diagnosis for cancer survivors [59]. To compare with the general population, mortality data of the general US population collected by the National Center for Health Statistics between 1975 and 2016 were also extracted from the SEER program [60].

As a publicly available database, the access of SEER data required a signed Research Data Agreement form. As it is considered as a non-human subject research and all information are anonymized, the data derived from SEER program are waived from IRB (Institutional Review Board) approval and informed consents.

### Definition of variables

Patients were followed up from the time of cancer diagnosis until death, the date of last follow-up, or exit at the end of the study (December 31, 2016). We examined the following variables for patients included in this study: age at diagnosis, sex, race, year of diagnosis, marital status, survival months, cause of death, anatomic site, cancer stage, and therapies including surgery, radiotherapy and chemotherapy.

Causes of death of patients with CRC were classified into three major groups: death from index cancer, death from other cancers and death from non-cancer causes. Causes of death were defined by SEER cause-specific death classification variable from death certificates [13]. Non-cancer causes were categorized into 26 major groups. These groups were further consolidated into 7 broad categories: infectious diseases, cardiovascular diseases, respiratory diseases, gastrointestinal and liver diseases, renal diseases, external injuries, and other causes. Although deaths from in situ, benign, or unknown behavior neoplasm were also classified to as non-cancer deaths by SEER program [13], these deaths were not considered as non-cancer deaths in our analyses.

In the SEER program, the survival duration of patients in the SEER database was measured in months and a month was the shortest time interval available for analysis. Any patient survival duration shorter than a month was recorded as 0 months. Thus, patients with a survival time coded as zero were converted to a-half month according to standard epidemiologic convention [61].

### Statistical analysis

Mortality rates were calculated as the number of deaths divided by the total person-years of follow-up. For non-cancer causes, the SMRs and 95% CIs of non-cancer deaths were calculated to perform a comparison with the general population [55, 61–63]. The SMR is based on the assumptions that the general population is cancer-free, and thus it can be used to compare the mortality rates from non-cancer diseases (e.g. cardiovascular, infectious and respiratory diseases) in the cancer population and the cancer-free population, which may reflect the impact of cancer and its treatments on non-cancer diseases. However, the SMR cannot be used for cancer-related causes. SMRs were estimated as the ratios of observed to expected number of deaths. The observed number of deaths represents the number of deaths from certain causes in cancer patients, and the expected number of deaths represents the number of people who died from the same causes in the general population with a similar distribution of age, sex, race and calendar year. For both age and calendar year, the value at diagnosis was used and were further divided into five-year categories in the course of standardization. 95% CI of SMR were obtained using an approximation from a Poisson distribution [61, 64].

For Objective 1, death data were extracted from SEER 9 registries database, which continually coded death trends from various causes by calendar year of death. For Objectives 2 and 3, we described the risk of death from non-cancer causes as a function of age at cancer diagnosis and follow-up time after cancer diagnosis, respectively. All statistical tests were two-sided, and P values less than 0.05 were considered to be statistically significant. Analyses were performed with SEER*Stat software version 8.3.6 and the R version 3.52 statistical software [58, 65].

## Supplementary Material

Supplementary Tables 1, 2 and 3

Supplementary Table 4

Supplementary Tables 5, 6 and 7

Supplementary Table 8
