# Supplementary material for: Cause of death among patients with colorectal cancer: a population-based study in the United States
Source: Aging (Albany NY). 2020 Nov 28;12(22):22927–48. doi: 10.18632/aging.104022 (PMC7746372; doi:10.18632/aging.104022)
Supplement: Supplementary Tables 1, 2 and 3 [file aging-12-104022-s001..pdf]

## SUPPLEMENTARY TABLES

**Supplementary Table 1.** Metastatic status of patients diagnosed with colorectal cancer between 2010 and 2016 in SEER 18 registries.

| Characteristics | No. of patients (%) | Person-years of follow-up | No. of deaths (%) | Non-cancer deaths | |
|---|---|---|---|---|---|
| | | | | No. of observed deaths (%) | SMR[1] (95% CI) |
| Liver metastasis | | | | | |
| Yes | 30,689 (14.8%) | 36,149 | 22,352 (34.2%) | 1,274 (8.5%) | 2.85 (2.70-3.02) |
| No | 177,070 (85.2%) | 469,716 | 42,957 (65.8%) | 13,727 (91.5%) | 1.61 (1.58-1.63) |
| Lung metastasis | | | | | |
| Yes | 10,407 (5.0%) | 10,549 | 7,925 (12.3%) | 430 (2.9%) | 2.92 (2.66-3.21) |
| No | 196,513 (95.0%) | 494,709 | 56,664 (87.7%) | 14,537 (97.1%) | 1.65 (1.62-1.67) |
| Bone metastasis | | | | | |
| Yes | 2,489 (1.2%) | 1,781 | 2,050 (3.1%) | 92 (0.6%) | 4.06 (3.31-4.98) |
| No | 204,461 (98.8%) | 503,430 | 62,541 (95.5%) | 14,855 (99.4%) | 1.66 (1.63-1.68) |
| Brain metastasis | | | | | |
| Yes | 576 (0.3%) | 368 | 487 (0.8%) | 27 (0.2%) | 7.07 (4.85-10.3) |
| No | 206,283 (99.7%) | 504,728 | 64,042 (99.2%) | 14,927 (99.8%) | 1.66 (1.64-1.69) |

**Supplementary Table 2.** Cancer-related deaths by liver metastasis in patients diagnosed with colorectal cancer between 2010 and 2016 in SEER 18 registries.

| Cancer-related cause of death | Liver metastasis | | | |
|---|---|---|---|---|
| | Yes | | No | |
| | Number | % | Number | % |
| Total | 21,078 | 100% | 29,230 | 100% |
| Colon and rectum | 19,413 | 92.1% | 25,581 | 87.5% |
| Other cancers | 1,665 | 7.90% | 3,649 | 12.48% |
| Lung and bronchus | 160 | 0.76% | 773 | 2.64% |
| Pancreas | 63 | 0.30% | 253 | 0.87% |
| In situ, benign or unknown behavior neoplasm | 56 | 0.27% | 152 | 0.52% |
| Liver | 214 | 1.02% | 133 | 0.46% |
| Other less frequent cancers | 1,172 | 5.56% | 2,338 | 8.00% |

**Supplementary Table 3. Cancer-related deaths by lung metastasis in patients diagnosed with colorectal cancer between 2010 and 2016 in SEER 18 registries.**

| Cancer-related cause of death | Lung metastasis | | | |
|---|---|---|---|---|
| | Yes | | No | |
| | Number | % | Number | % |
| Total | 7,495 | 100% | 42,127 | 100% |
| Colon and rectum | 6,829 | 91.1% | 37,564 | 89.2% |
| Other cancers | 666 | 8.9% | 4,563 | 10.8% |
| Lung and Bronchus | 154 | 2.1% | 777 | 1.8% |
| Liver | 39 | 0.5% | 297 | 0.7% |
| Pancreas | 23 | 0.3% | 288 | 0.7% |
| Other Digestive Organs | 20 | 0.3% | 190 | 0.5% |
| Anus | 19 | 0.3% | 121 | 0.3% |
| In situ, benign or unknown behavior neoplasm | 13 | 0.2% | 195 | 0.5% |
| Other less frequent cancers | 398 | 5.3% | 2,695 | 6.4% |
