# Supplementary material for: Cause of death among patients with colorectal cancer: a population-based study in the United States
Source: Aging (Albany NY). 2020 Nov 28;12(22):22927–48. doi: 10.18632/aging.104022 (PMC7746372; doi:10.18632/aging.104022)
Supplement: Supplementary Tables 5, 6 and 7 [file aging-12-104022-s003..pdf]

SUPPLEMENTARY TABLES

Supplementary Table 5. Cause of death for CRC patients between 1975 and 2016 in SEER 18 registries by surgical operation.

Cause of death	Surgery					
	No			Yes		
	No. of observed deaths (%)	Mortality rates (per 100,000 person-years)	SMR[1,2] (95% CI)	No. of observed deaths (%)	Mortality rates (per 100,000 person-years)	SMR[1,2] (95% CI)
All causes of death	92,843 (100%)	48,916.4		431,581 (100%)	8,915.2	
Colorectal cancer	68,165 (73.4%)	35,914.2		199,787 (46.3%)	4,127.0	
Other cancers	8,549 (9.2%)	4,504.2		45,514 (10.5%)	940.2	
Non-cancer causes	16,129 (17.4%)	8,497.9	3.48 (3.43-3.53)	186,280 (43.2%)	3,848.0	1.95 (1.94-1.96)
Infectious diseases	219 (0.2%)	115.4	3.81 (3.34-4.35)	2,590 (0.6%)	53.5	2.78 (2.67-2.89)
Pneumonia and influenza	645 (0.7%)	339.8	3.31 (3.06-3.57)	8,071 (1.9%)	166.7	2.15 (2.10-2.19)
Syphilis	0 (0.0%)	0.0	0.00 (0.00-NA)	2 (0.0005%)	0.0	0.80 (0.20-3.20)
Tuberculosis	9 (0.01%)	4.7	3.15 (1.64-6.06)	66 (0.02%)	1.4	0.87 (0.68-1.10)
Septicemia	430 (0.5%)	226.6	5.65 (5.14-6.21)	3,739 (0.9%)	77.2	2.63 (2.55-2.72)
Other infectious and parasitic diseases including HIV	231 (0.2%)	121.7	7.32 (6.44-8.33)	1,617 (0.4%)	33.4	2.59 (2.47-2.72)
Cardiovascular diseases	8,681 (9.4%)	4,573.8	3.41 (3.34-3.48)	98,526 (22.8%)	2,035.3	1.80 (1.78-1.81)
Diseases of heart	6,871 (7.4%)	3,620.1	3.56 (3.48-3.65)	75,372 (17.5%)	1,557.0	1.78 (1.77-1.80)
Hypertension without heart disease	12 (0.01%)	6.3	1.39 (0.79-2.45)	156 (0.04%)	3.2	0.72 (0.61-0.84)
Aortic aneurysm and dissection	107 (0.1%)	56.4	2.90 (2.40-3.51)	1,360 (0.3%)	28.1	1.42 (1.35-1.50)
Atherosclerosis	186 (0.2%)	98.0	3.83 (3.32-4.42)	1,758 (0.4%)	36.3	1.82 (1.73-1.90)
Cerebrovascular diseases	1,215 (1.3%)	640.1	2.70 (2.56-2.86)	16,413 (3.8%)	339.0	1.79 (1.76-1.81)
Other diseases of arteries, arterioles, capillaries	83 (0.1%)	43.7	3.18 (2.56-3.94)	1,033 (0.2%)	21.3	1.89 (1.78-2.01)
Respiratory diseases	1,144 (1.2%)	602.7	3.83 (3.61-4.05)	12,472 (2.9%)	257.6	1.86 (1.83-1.90)
Chronic obstructive pulmonary disease and allied cond	1,144 (1.2%)	602.7	3.83 (3.61-4.05)	12,472 (2.9%)	257.6	1.86 (1.83-1.90)
Gastrointestinal diseases	459 (0.5%)	241.8	2.49 (2.27-2.73)	6,134 (1.4%)	126.7	1.51 (1.48-1.55)
Stomach and duodenal ulcers	42 (0.0%)	22.1	3.41 (2.52-4.62)	572 (0.1%)	11.8	1.90 (1.75-2.06)
Chronic liver disease and cirrhosis	254 (0.3%)	133.8	5.23 (4.62-5.91)	2,018 (0.5%)	41.7	1.50 (1.43-1.56)
Renal diseases	388 (0.4%)	204.4	3.74 (3.38-4.13)	4,494 (1.0%)	92.8	2.40 (2.33-2.47)
Nephritis, nephrotic syndrome and nephrosis	388 (0.4%)	204.4	3.74 (3.38-4.13)	4,494 (1.0%)	92.8	2.40 (2.33-2.47)
External injuries	1,315 (1.4%)	692.8	4.30 (4.07-4.54)	13,495 (3.1%)	278.8	2.29 (2.25-2.33)
Accidents and adverse effects	293 (0.3%)	154.4	2.03 (1.81-2.28)	4,786 (1.1%)	98.9	1.59 (1.55-1.64)
Suicide and self-inflicted injury	154 (0.2%)	81.1	4.85 (4.14-5.67)	1,192 (0.3%)	24.6	1.45 (1.37-1.53)
Homicide and legal intervention	296 (0.3%)	156.0	4.86 (4.34-5.45)	2,590 (0.6%)	53.5	1.57 (1.51-1.63)
Other non-cancer causes	3,846 (4.1%)	2,026.3	3.39 (3.28-3.49)	48,569 (11.3%)	1,003.3	2.38 (2.36-2.41)
Alzheimer's disease	409 (0.4%)	215.5	2.51 (2.28-2.77)	7,100 (1.6%)	146.7	3.15 (3.07-3.22)
Diabetes mellitus	487 (0.5%)	256.6	2.97 (2.72-3.25)	6,711 (1.6%)	138.6	1.92 (1.88-1.97)
Congenital anomalies	9 (0.01%)	4.7	1.79 (0.93-3.44)	163 (0.04%)	3.4	1.31 (1.13-1.53)
Certain conditions originating in perinatal period	1 (0.001%)	0.5	2,806.2 (395.3-19,921.9)	6 (0.001%)	0.1	107.5 (48.3-239.2)
Complications of pregnancy,	10 (0.01%)	5.3	106.3 (57.2-	23 (0.01%)	0.5	15.3 (10.2-23.1)

childbirth, puerperium			197.5)			
Symptoms, signs and ill-defined conditions	227 (0.2%)	119.6	3.41 (2.99-3.88)	2,333 (0.5%)	48.2	1.92 (1.84-2.00)
Other cause of death	2,703 (2.9%)	1,424.1	3.67 (3.53-3.81)	32,233 (7.5%)	665.8	2.43 (2.40-2.45)

Abbreviations: SMR, standardized mortality ratios; CI, confidence interval.

[1]The calculating of SMR is based on the hypothesis that the general population is cancer-free, thus the expected deaths and SMR for colorectal cancer and other cancers cannot be calculated.

[2]The SMRs were estimated as the ratios of observed to expected number of deaths. The observed values represented the number of deaths in cancer patients, whereas the expected values represented the number of individuals who died of the same causes in the general population, with a similar distribution of age, sex, race, and calendar year.

Supplementary Table 6. Causes of death for CRC patients between 1975 and 2016 in SEER 18 registries by chemotherapy.

Cause of death	Chemotherapy					
	Yes			No/Unknown		
	No. of observed deaths (%)	Mortality rates (per 100,000 person-years)	SMR[1,2] (95% CI)	No. of observed deaths (%)	Mortality rates (per 100,000 person-years)	SMR[1,2] (95% CI)
All causes of death	139,652 (100%)	11,492.3		391,855 (100%)	10,202.4	
Colorectal cancer	104,870 (75.1%)	8,630.0		168,031 (42.9%)	4,374.9	
Other cancers	11,193 (8.0%)	921.1		43,423 (11.1%)	1,130.6	
Non-cancer causes	23,589 (16.9%)	1,941.2	1.63 (1.61-1.65)	180,401 (46.0%)	4,696.9	2.09 (2.09-2.10)
Infectious diseases	1,841 (1.3%)	151.5	2.12 (2.03-2.22)	13,101 (3.3%)	341.1	2.44 (2.40-2.49)
Pneumonia and influenza	793 (0.6%)	65.3	1.72 (1.61-1.85)	7,999 (2.0%)	208.3	2.27 (2.22-2.32)
Syphilis	0 (0.0%)	0.0	NA	2 (0.001%)	0.1	0.82 (0.21-3.29)
Tuberculosis	12 (0.01%)	1.0	1.11 (0.63-1.95)	63 (0.02%)	1.6	0.92 (0.72-1.17)
Septicemia	690 (0.5%)	56.8	2.68 (2.49-2.89)	3,517 (0.9%)	91.6	2.82 (2.73-2.91)
Other infectious and parasitic diseases including HIV	133 (0.1%)	10.9	1.63 (1.37-1.93)	997 (0.3%)	26.0	2.02 (1.90-2.15)
Cardiovascular diseases	11,175 (8.0%)	919.6	1.48 (1.45-1.51)	96,911 (24.7%)	2,523.2	1.93 (1.92-1.94)
Diseases of heart	8,672 (6.2%)	713.6	1.47 (1.44-1.50)	74,275 (19.0%)	1,933.8	1.93 (1.91-1.94)
Hypertension without heart disease	344 (0.2%)	28.3	2.23 (2.00-2.48)	2,476 (0.6%)	64.5	2.94 (2.83-3.06)
Aortic aneurysm and dissection	129 (0.1%)	10.6	0.86 (0.73-1.03)	1,349 (0.3%)	35.1	1.59 (1.51-1.68)
Atherosclerosis	135 (0.1%)	11.1	1.62 (1.37-1.91)	1,826 (0.5%)	47.5	1.94 (1.85-2.03)
Cerebrovascular diseases	1,762 (1.3%)	145.0	1.49 (1.42-1.56)	15,988 (4.1%)	416.3	1.88 (1.85-1.91)
Other diseases of arteries, arterioles, capillaries	4,560 (3.3%)	375.3	2.09 (2.03-2.15)	30,639 (7.8%)	797.7	2.57 (2.54-2.60)
Respiratory diseases	1,641 (1.2%)	135.0	1.36 (1.30-1.43)	12,072 (3.1%)	314.3	2.07 (2.03-2.11)
Chronic obstructive pulmonary disease and allied cond	1,641 (1.2%)	135.0	1.36 (1.30-1.43)	12,072 (3.1%)	314.3	2.07 (2.03-2.11)
Gastrointestinal diseases	376 (0.3%)	30.9	1.10 (1.00-1.22)	2,531 (0.6%)	65.9	1.84 (1.77-1.91)
Stomach and duodenal ulcers	82 (0.1%)	6.7	2.10 (1.69-2.61)	536 (0.1%)	14.0	1.94 (1.78-2.11)
Chronic liver disease and cirrhosis	294 (0.2%)	24.2	0.97 (0.87-1.09)	1,995 (0.5%)	51.9	1.81 (1.73-1.89)
Renal diseases	591 (0.4%)	48.6	1.85 (1.70-2.00)	4,328 (1.1%)	112.7	2.59 (2.52-2.67)
Nephritis, nephrotic syndrome and nephrosis	591 (0.4%)	48.6	1.85 (1.70-2.00)	4,328 (1.1%)	112.7	2.59 (2.52-2.67)
External injuries	1,183 (0.8%)	97.4	1.39 (1.31-1.47)	5,463 (1.4%)	142.2	1.60 (1.56-1.65)
Accidents and adverse effects	828 (0.6%)	68.1	1.38 (1.29-1.48)	4,290 (1.1%)	111.7	1.67 (1.62-1.72)
Suicide and self-inflicted injury	318 (0.2%)	26.2	1.59 (1.42-1.77)	1,042 (0.3%)	27.1	1.58 (1.49-1.68)

Homicide and legal intervention	37 (0.03%)	3.0	0.70 (0.51-0.96)	131 (0.0%)	3.4	0.75 (0.63-0.89)
Other non-cancer causes	346 (0.2%)	28.5	2.49 (2.24-2.76)	1,520 (0.4%)	39.6	2.92 (2.78-3.07)
Alzheimer's disease	813 (0.6%)	66.9	2.68 (2.50-2.87)	6,720 (1.7%)	175.0	3.16 (3.08-3.23)
Diabetes mellitus	960 (0.7%)	79.0	1.44 (1.35-1.53)	6,289 (1.6%)	163.7	2.09 (2.04-2.14)
Congenital anomalies	18 (0.01%)	1.5	0.68 (0.43-1.07)	156 (0.04%)	4.1	1.51 (1.29-1.77)
Certain conditions originating in perinatal period	0 (0.0%)	0.0	NA	7 (0.002%)	0.2	216.0 (103.0-453.0)
Complications of pregnancy, childbirth, puerperium	20 (0.01%)	1.6	31.6 (20.4-49.0)	13 (0.003%)	0.3	13.4 (7.77-23.0)
Symptoms, signs and ill-defined conditions	411 (0.3%)	33.8	2.33 (2.11-2.57)	2,171 (0.6%)	56.5	1.95 (1.87-2.03)
Other cause of death	6,782 (4.9%)	558.1	2.02 (1.97-2.07)	45,995 (11.7%)	1,197.5	2.52 (2.49-2.54)

Abbreviations: SMR, standardized mortality ratios; CI, confidence interval.
[1]The calculating of SMR is based on the hypothesis that the general population is cancer-free, thus the expected deaths and SMR for colorectal cancer and other cancers cannot be calculated.
[2]The SMRs were estimated as the ratios of observed to expected number of deaths. The observed values represented the number of deaths in cancer patients, whereas the expected values represented the number of individuals who died of the same causes in the general population, with a similar distribution of age, sex, race, and calendar year.

Supplementary Table 7. Causes of death for CRC patients between 1975 and 2016 in SEER 18 registries by radiotherapy.

Cause of death	Radiotherapy					
	No/unknown			Yes		
	No. of observed deaths (%)	Mortality rates (per 100,000 person-years)	SMR[1,2] (95% CI)	No. of observed deaths (%)	Mortality rates (per 100,000 person-years)	SMR[1,2] (95% CI)
All causes of death	473,615 (100.0%)	10507.2		57,892 (100.0%)	10554.9	
Colorectal cancer	233,671 (49.3%)	5184.0		39,230 (67.8%)	7152.5	
Other cancers	48,996 (10.3%)	1087.0		5,620 (9.7%)	1024.6	
Non-cancer causes	190,948 (40.3%)	4236.2	2.04 (2.03-2.05)	13,042 (22.5%)	2377.8	1.85 (1.82-1.88)
Infectious diseases	13,908 (2.9%)	308.6	2.39 (2.35-2.43)	1,034 (1.8%)	188.5	2.49 (2.34-2.65)
Pneumonia and influenza	8,321 (1.8%)	184.6	2.22 (2.18-2.27)	471 (0.8%)	85.9	2.00 (1.83-2.19)
Syphilis	2 (0.0004%)	0.04	0.80 (0.20-3.21)	0 (0.0%)	0.0	NA
Tuberculosis	69 (0.01%)	1.5	0.93 (0.74-1.18)	6 (0.01%)	1.1	1.06 (0.48-2.36)
Septicemia	3,833 (0.8%)	85.0	2.75 (2.67-2.84)	374 (0.6%)	68.2	3.32 (3.00-3.67)
Other infectious and parasitic diseases including HIV	1,683 (0.4%)	37.3	2.81 (2.68-2.95)	183 (0.3%)	33.4	3.00 (2.60-3.47)
Cardiovascular diseases	101,742 (21.5%)	2257.2	1.89 (1.88-1.90)	6,344 (11.0%)	1156.6	1.66 (1.62-1.70)
Diseases of heart	77,979 (16.5%)	1730.0	1.88 (1.87-1.89)	4,968 (8.6%)	905.8	1.65 (1.60-1.69)
Hypertension without heart disease	2,631 (0.6%)	58.4	2.84 (2.73-2.95)	189 (0.3%)	34.5	2.78 (2.41-3.20)
Aortic aneurysm and dissection	1,397 (0.3%)	31.0	1.52 (1.44-1.60)	81 (0.1%)	14.8	1.08 (0.87-1.34)
Atherosclerosis	1,875 (0.4%)	41.6	1.92 (1.84-2.01)	86 (0.1%)	15.7	1.73 (1.40-2.14)
Cerebrovascular diseases	16,827 (3.6%)	373.3	1.85 (1.82-1.88)	923 (1.6%)	168.3	1.58 (1.48-1.69)
Other diseases of arteries, arterioles, capillaries	1,033 (0.2%)	22.9	1.93 (1.81-2.05)	97 (0.2%)	17.7	2.50 (2.05-3.05)
Respiratory diseases	12,782 (2.7%)	283.6	1.97 (1.94-2.01)	931 (1.6%)	169.7	1.71 (1.60-1.82)
Chronic obstructive pulmonary disease and allied cond	12,782 (2.7%)	283.6	1.97 (1.94-2.01)	931 (1.6%)	169.7	1.71 (1.60-1.82)
Gastrointestinal diseases	2,709 (0.6%)	60.1	1.74 (1.68-1.81)	198 (0.3%)	36.1	1.19 (1.04-1.37)
Stomach and duodenal ulcers	582 (0.1%)	12.9	1.98 (1.82-2.15)	36 (0.1%)	6.6	1.74 (1.26-2.41)
Chronic liver disease and cirrhosis	2,127 (0.4%)	47.2	1.69 (1.62-1.76)	162 (0.3%)	29.5	1.11 (0.95-1.30)
Renal diseases	4,611 (1.0%)	102.3	2.50 (2.43-2.57)	308 (0.5%)	56.2	2.16 (1.93-2.42)
Nephritis, nephrotic syndrome and nephrosis	4,611 (1.0%)	102.3	2.50 (2.43-2.57)	308 (0.5%)	56.2	2.16 (1.93-2.42)
External injuries	6,044 (1.3%)	134.1	1.57 (1.53-1.61)	602 (1.0%)	109.8	1.47 (1.35-1.59)
Accidents and adverse effects	4,720 (1.0%)	104.7	1.64 (1.59-1.68)	398 (0.7%)	72.6	1.39 (1.26-1.54)
Suicide and self-inflicted injury	1,173 (0.2%)	26.0	1.54 (1.46-1.63)	187 (0.3%)	34.1	1.89 (1.64-2.18)
Homicide and legal intervention	151 (0.03%)	3.3	0.75 (0.64-0.88)	17 (0.03%)	3.1	0.66 (0.41-1.06)
Other non-cancer causes	49,152 (10.4%)	1090.4	2.45 (2.42-2.47)	3,625 (6.3%)	660.9	2.36 (2.29-2.44)
Alzheimer's disease	7,141 (1.5%)	158.4	3.11 (3.04-3.18)	392 (0.7%)	71.5	2.84 (2.57-3.14)
Diabetes mellitus	6,741 (1.4%)	149.6	1.99 (1.94-2.04)	508 (0.9%)	92.6	1.74 (1.59-1.90)
Congenital anomalies	168 (0.04%)	3.7	1.43 (1.23-1.66)	6 (0.01%)	1.1	0.49 (0.22-1.09)
Certain conditions originating in	7 (0.001%)	0.2	127.2 (60.6-266.8)	0 (0.0%)	0.0	NA

perinatal period						
Complications of pregnancy, childbirth, puerperium	31 (0.01%)	0.7	22.5 (15.9-32.1)	2 (0.003%)	0.4	8.75 (2.19-35.0)
Symptoms, signs and ill-defined conditions	2,366 (0.5%)	52.5	1.97 (1.89-2.05)	216 (0.4%)	39.4	2.44 (2.13-2.78)
Other cause of death	32,698 (6.9%)	725.4	2.50 (2.47-2.53)	2,501 (4.3%)	456.0	2.49 (2.40-2.59)

Abbreviations: SMR, standardized mortality ratios; CI, confidence interval.

[1]The calculating of SMR is based on the hypothesis that the general population is cancer-free, thus the expected deaths and SMR for colorectal cancer and other cancers cannot be calculated.

[2]The SMRs were estimated as the ratios of observed to expected number of deaths. The observed values represented the number of deaths in cancer patients, whereas the expected values represented the number of individuals who died of the same causes in the general population, with a similar distribution of age, sex, race, and calendar year.
