# Supplementary material for: Cause of death among patients with colorectal cancer: a population-based study in the United States
Source: Aging (Albany NY). 2020 Nov 28;12(22):22927–48. doi: 10.18632/aging.104022 (PMC7746372; doi:10.18632/aging.104022)
Supplement: Supplementary Table 8 [file aging-12-104022-s004..docx]

**Supplementary Table 8** Causes of death among patients diagnosed with colorectal cancer between 1975 and 2016 in SEER 18 registries by time from cancer diagnosis.

| Causes of death | Time from cancer diagnosis | | | | | | | | | | | | | |
| --- | --- | --- | --- | --- | --- | --- | --- | --- | --- | --- | --- | --- | --- | --- |
|  | With 1 year | |  | 1-5 years | |  | 5-10 years | |  | 10-15 years | |  | 15+ years | |
|  | No. of observed deaths (%) | SMR^1,2^ (95% CI) |  | No. of observed deaths (%) | SMR^1,2^ (95% CI) |  | No. of observed deaths (%) | SMR^1,2^ (95% CI) |  | No. of observed deaths (%) | SMR^1,2^ (95% CI) |  | No. of observed deaths (%) | SMR^1,2^ (95% CI) |
| All causes of death | 167,094 (100%) | NA |  | 195,087 (100%) | NA |  | 86,205 (100%) | NA |  | 42,766 (100%) | NA |  | 40,355 (100%) | NA |
| Colon and rectum cancer | 117,584 (70.4%) | NA |  | 125,142 (64.1%) | NA |  | 23,846 (27.7%) | NA |  | 4,206 (9.8%) | NA |  | 2,123 (5.3%) | NA |
| Other cancers | 11,482 (6.9%) | NA |  | 16,716 (8.6%) | NA |  | 12,641 (14.7%) | NA |  | 7,189 (16.8%) | NA |  | 6,588 (16.3%) | NA |
| Non-cancer causes | 38,028 (22.8%) | 2.24 (2.22-2.27) |  | 53,229 (27.3%) | 1.22 (1.21-1.23) |  | 49,718 (57.7%) | 1.97 (1.95-1.99) |  | 31,371 (73.4%) | 3.16 (3.12-3.19) |  | 31,644 (78.4%) | 6.32 (6.25-6.39) |
| Infectious diseases | 3,504 (2.1%) | 3.12 (3.02-3.23) |  | 3,837 (2.0%) | 1.34 (1.30-1.38) |  | 3,459 (4.0%) | 2.15 (2.08-2.22) |  | 2,104 (4.9%) | 3.53 (3.39-3.69) |  | 2,038 (5.1%) | 7.79 (7.46-8.14) |
| Pneumonia and influenza | 1,573 (0.9%) | 2.20 (2.09-2.31) |  | 2,192 (1.1%) | 1.22 (1.17-1.28) |  | 2,243 (2.6%) | 2.29 (2.20-2.39) |  | 1,430 (3.3%) | 4.11 (3.90-4.33) |  | 1,354 (3.4%) | 9.23 (8.75-9.73) |
| Syphilis | 0 (0.0%) | NA |  | 1 (0.001%) | 0.96 (0.14-6.83) |  | 1 (0.001%) | 1.58 (0.22-11.2) |  | 0 (0.0%) | NA |  | 0 (0.0%) | NA |
| Tuberculosis | 16 (0.01%) | 1.45 (0.89-2.37) |  | 24 (0.01%) | 0.80 (0.53-1.19) |  | 15 (0.02%) | 0.74 (0.45-1.23) |  | 11 (0.03%) | 1.06 (0.59-1.92) |  | 9 (0.02%) | 1.15 (0.60-2.22) |
| Septicemia | 1,326 (0.8%) | 5.08 (4.81-5.36) |  | 1,105 (0.6%) | 1.64 (1.54-1.73) |  | 828 (1.0%) | 2.17 (2.02-2.32) |  | 468 (1.1%) | 3.43 (3.14-3.76) |  | 480 (1.2%) | 9.59 (8.77-10.5) |
| Other infectious and parasitic diseases including HIV | 589 (0.4%) | 5.48 (5.05-5.94) |  | 515 (0.3%) | 1.80 (1.65-1.96) |  | 372 (0.4%) | 2.21 (1.99-2.44) |  | 195 (0.5%) | 2.98 (2.59-3.43) |  | 195 (0.5%) | 6.16 (5.35-7.09) |
| Cardiovascular diseases | 20,817 (12.5%) | 2.18 (2.15-2.21) |  | 29,219 (15.0%) | 1.18 (1.17-1.20) |  | 26,368 (30.6%) | 1.81 (1.79-1.83) |  | 16,071 (37.6%) | 2.72 (2.68-2.76) |  | 15,611 (38.7%) | 5.10 (5.02-5.18) |
| Diseases of heart | 16,478 (9.9%) | 2.27 (2.24-2.30) |  | 22,476 (11.5%) | 1.19 (1.18-1.21) |  | 20,117 (23.3%) | 1.79 (1.77-1.82) |  | 12,155 (28.4%) | 2.62 (2.58-2.67) |  | 11,721 (29.0%) | 4.72 (4.63-4.80) |
| Hypertension without heart disease | 408 (0.2%) | 2.17 (1.97-2.39) |  | 662 (0.3%) | 1.43 (1.33-1.55) |  | 724 (0.8%) | 3.02 (2.81-3.25) |  | 499 (1.2%) | 6.38 (5.84-6.96) |  | 527 (1.3%) | 18.8 (17.2-20.4) |
| Aortic aneurysm and dissection | 334 (0.2%) | 2.24 (2.01-2.49) |  | 398 (0.2%) | 0.97 (0.88-1.08) |  | 342 (0.4%) | 1.30 (1.17-1.45) |  | 208 (0.5%) | 1.78 (1.56-2.04) |  | 196 (0.5%) | 3.27 (2.84-3.76) |
| Atherosclerosis | 400 (0.2%) | 2.14 (1.94-2.36) |  | 581 (0.3%) | 1.27 (1.17-1.37) |  | 436 (0.5%) | 1.73 (1.58-1.90) |  | 286 (0.7%) | 3.19 (2.84-3.58) |  | 258 (0.6%) | 7.08 (6.26-7.99) |
| Cerebrovascular diseases | 2,953 (1.8%) | 1.78 (1.72-1.84) |  | 4,825 (2.5%) | 1.14 (1.11-1.17) |  | 4,485 (5.2%) | 1.84 (1.79-1.90) |  | 2,749 (6.4%) | 2.95 (2.84-3.06) |  | 2,738 (6.8%) | 6.45 (6.21-6.69) |
| Other diseases of arteries, arterioles, capillaries | 244 (0.1%) | 2.56 (2.26-2.91) |  | 277 (0.1%) | 1.12 (1.00-1.26) |  | 264 (0.3%) | 1.82 (1.61-2.05) |  | 174 (0.4%) | 2.98 (2.57-3.46) |  | 171 (0.4%) | 5.91 (5.09-6.86) |
| Respiratory diseases | 2,477 (1.5%) | 2.17 (2.09-2.26) |  | 3,870 (2.0%) | 1.27 (1.23-1.31) |  | 3,500 (4.1%) | 1.93 (1.87-1.99) |  | 2,013 (4.7%) | 2.80 (2.68-2.93) |  | 1,853 (4.6%) | 5.86 (5.60-6.13) |
| Chronic obstructive pulmonary disease and allied cond | 2,477 (1.5%) | 2.17 (2.09-2.26) |  | 3,870 (2.0%) | 1.27 (1.23-1.31) |  | 3,500 (4.1%) | 1.93 (1.87-1.99) |  | 2,013 (4.7%) | 2.80 (2.68-2.93) |  | 1,853 (4.6%) | 5.86 (5.60-6.13) |
| Gastrointestinal | 836 (0.5%) | 3.65 (3.41-3.90) |  | 827 (0.4%) | 1.27 (1.18-1.36) |  | 668 (0.8%) | 1.50 (1.39-1.62) |  | 330 (0.8%) | 1.47 (1.32-1.64) |  | 246 (0.6%) | 1.46 (1.29-1.65) |
| Stomach and duodenal ulcers | 150 (0.1%) | 3.06 (2.61-3.59) |  | 160 (0.1%) | 1.23 (1.06-1.44) |  | 149 (0.2%) | 1.85 (1.58-2.18) |  | 74 (0.2%) | 2.10 (1.67-2.64) |  | 85 (0.2%) | 4.16 (3.36-5.14) |
| Chronic liver disease and cirrhosis | 686 (0.4%) | 3.81 (3.53-4.10) |  | 667 (0.3%) | 1.28 (1.18-1.38) |  | 519 (0.6%) | 1.42 (1.31-1.55) |  | 256 (0.6%) | 1.35 (1.20-1.53) |  | 161 (0.4%) | 1.09 (0.93-1.27) |
| Renal diseases | 896 (0.5%) | 2.52 (2.36-2.69) |  | 1,326 (0.7%) | 1.47 (1.39-1.55) |  | 1,237 (1.4%) | 2.49 (2.36-2.64) |  | 694 (1.6%) | 4.07 (3.78-4.38) |  | 766 (1.9%) | 12.0 (11.2-12.9) |
| Nephritis, nephrotic syndrome and nephrosis | 896 (0.5%) | 2.52 (2.36-2.69) |  | 1,326 (0.7%) | 1.47 (1.39-1.55) |  | 1,237 (1.4%) | 2.49 (2.36-2.64) |  | 694 (1.6%) | 4.07 (3.78-4.38) |  | 766 (1.9%) | 12.0 (11.2-12.9) |
| External injuries | 999 (0.6%) | 1.54 (1.45-1.64) |  | 2,062 (1.1%) | 1.18 (1.13-1.24) |  | 1,602 (1.9%) | 1.50 (1.43-1.57) |  | 1,001 (2.3%) | 2.11 (1.98-2.24) |  | 982 (2.4%) | 3.02 (2.84-3.21) |
| Accidents and adverse effects | 707 (0.4%) | 1.40 (1.30-1.51) |  | 1,453 (0.7%) | 1.09 (1.04-1.15) |  | 1,274 (1.5%) | 1.61 (1.53-1.70) |  | 824 (1.9%) | 2.48 (2.31-2.65) |  | 860 (2.1%) | 4.11 (3.84-4.39) |
| Suicide and self-inflicted injury | 273 (0.2%) | 2.36 (2.10-2.66) |  | 538 (0.3%) | 1.63 (1.50-1.78) |  | 288 (0.3%) | 1.30 (1.16-1.46) |  | 151 (0.4%) | 1.38 (1.18-1.62) |  | 110 (0.3%) | 1.32 (1.09-1.59) |
| Homicide and legal intervention | 19 (0.01%) | 0.71 (0.45-1.11) |  | 71 (0.04%) | 0.91 (0.72-1.14) |  | 40 (0.05%) | 0.70 (0.51-0.95) |  | 26 (0.1%) | 0.79 (0.54-1.16) |  | 12 (0.03%) | 0.37 (0.21-0.65) |
| Other non-cancer causes | 8,499 (5.1%) | 2.17 (2.12-2.22) |  | 12,088 (6.2%) | 1.23 (1.21-1.26) |  | 12,884 (14.9%) | 2.45 (2.41-2.50) |  | 9,158 (21.4%) | 4.95 (4.85-5.05) |  | 10,148 (25.1%) | 12.6 (12.3-12.8) |
| Alzheimer’s disease | 385 (0.2%) | 0.73 (0.66-0.81) |  | 1,303 (0.7%) | 1.07 (1.01-1.13) |  | 2,084 (2.4%) | 3.87 (3.71-4.04) |  | 1,714 (4.0%) | 13.8 (13.2-14.5) |  | 2,047 (5.1%) | 93.9 (90.0-98.1) |
| Diabetes mellitus | 1,124 (0.7%) | 1.94 (1.83-2.06) |  | 2,083 (1.1%) | 1.34 (1.28-1.40) |  | 1,848 (2.1%) | 1.92 (1.84-2.01) |  | 1,189 (2.8%) | 2.99 (2.83-3.17) |  | 1,005 (2.5%) | 5.50 (5.17-5.85) |
| Congenital anomalies | 32 (0.02%) | 1.76 (1.25-2.49) |  | 37 (0.02%) | 0.73 (0.53-1.00) |  | 41 (0.05%) | 1.21 (0.89-1.64) |  | 26 (0.1%) | 1.60 (1.09-2.35) |  | 38 (0.1%) | 3.64 (2.65-5.01) |
| Certain conditions originating in perinatal period | 2 (0.001%) | 387.4 (96.9-1548.9) |  | 1 (0.001%) | 47.1 (6.6-334.1) |  | 1 (0.001%) | 44.9 (6.33-319.0) |  | 0 (0.0%) | NA |  | 3 (0.01%) | 6295.0 (2030.2-19518.6) |
| Complications of pregnancy, childbirth, puerperium | 13 (0.01%) | 51.2 (29.7-88.2) |  | 15 (0.01%) | 22.1 (13.3-36.7) |  | 2 (0.002%) | 5.61 (1.40-22.4) |  | 2 (0.005%) | 12.9 (3.23-51.6) |  | 1 (0.002%) | 6.23 (0.88-44.2) |
| Symptoms, signs and ill-defined conditions | 452 (0.3%) | 1.97 (1.80-2.16) |  | 654 (0.3%) | 1.15 (1.07-1.24) |  | 601 (0.7%) | 1.95 (1.80-2.11) |  | 388 (0.9%) | 3.31 (3.00-3.66) |  | 487 (1.2%) | 7.12 (6.52-7.79) |
| Other cause of death | 6,491 (3.9%) | 2.53 (2.47-2.59) |  | 7,995 (4.1%) | 1.25 (1.22-1.28) |  | 8,307 (9.6%) | 2.44 (2.39-2.49) |  | 5,839 (13.7%) | 4.88 (4.76-5.01) |  | 6,567 (16.3%) | 12.6 (12.3-12.9) |

Abbreviations: SMR, standardized mortality ratios; CI, confidence interval.

^1^ The calculating of SMR is based on the hypothesis that the general population is cancer-free, thus the expected deaths and SMR for colorectal cancer and other cancers cannot be calculated.

^2^ The SMRs were estimated as the ratios of observed to expected number of deaths. The observed values represented the number of deaths in cancer patients, whereas the expected values represented the number of individuals who died of the same causes in the general population, with a similar distribution of age, sex, race, and calendar year
